# Network analysis of anxiety, depression and insomnia in the elderly in Jiangsu Province

**DOI:** 10.7717/peerj.20868

**Published:** 2026-03-31

**Authors:** Wantong Han, Hualing Chen, Mingwang Fu, Haoran Zhou, Jinshui Xu, Can Zhao, Xiang Lu, Hou-Guang Zhou, Zheng-Kai Shen, Bingwei Chen

**Affiliations:** 1 Department of Epidemiology and Biostatistics, Southeast University, Nanjing, Jiangsu, China; 2 Jiangsu Provincial Center for Disease Control and Prevention, Nanjing, China; 3 Department of Geriatrics, Sir Run Run Hospital, Nanjing Medical University, Nanjing, Jiangsu, China; 4 Key Laboratory for Aging &Disease, Nanjing Medical University, Nanjing, Jiangsu, China; 5 Department of Geriatrics of Huashan Hospital, National Clinical Research Center for Aging and Medicine, Fudan University, Shanghai, China; 6 Key Laboratory of Environmental Medicine Engineering, Ministry of Education, School of Public Health, Southeast University, Nanjing, China

**Keywords:** Insomnia, Depression, Anxiety, Elderly, Network analysis

## Abstract

**Objectives:**

Mental health is an important indicator in the evaluation of healthy aging. As individuals age, they become increasingly susceptible to symptoms of anxiety, depression, and insomnia, which are often exacerbated by diminished daily functioning, reduced social engagement, and a decline in self-care capabilities. This study uses network analysis to examine the symptom interactions and applies clique percolation to detect overlapping items across the three symptom domains.

**Methods:**

This online survey was conducted in three cities in Jiangsu Province between September 2023 and March 2024. Using a cross-sectional design, anxiety, depression, and insomnia symptoms in elderly individuals were assessed with the Generalized Anxiety Disorder Scale (GAD-7), the Patient Health Questionnaire (PHQ-9), and the Insomnia Severity Index (ISI-7). The symptom network was constructed using the EBICglasso algorithm and the clique-percolation algorithm was employed to reveal cross-dimensional connections between symptoms. The study was approved by the Ethics Committee of Huashan Hospital, Fudan University (Approval No. 2022-057).

**Results:**

A total of 2,086 elderly individuals were included, with mean age of 72.5 years and 1,061 females (50.9%). The prevalence rates for anxiety, depression, and insomnia were 21.6%, 13.2%, and 46.3%, respectively. Based on clique percolation analysis, three symptom clusters were identified, revealing clear cross-dimensional symptom connectivity among anxiety, depression, and insomnia. Network analysis revealed that the central symptoms—namely “difficulty maintaining sleep,” “the perceived impact of sleep disturbances on quality of life by others,” and “sleep interference with daytime functioning”—were pivotal in the network. The bridging nodes linking anxiety, depression, and insomnia were identified as “sleep problems,” “suicidal ideation,” and “restlessness.”

**Conclusion:**

The central nodes within this network are predominantly sleep-related, underscoring their significant role in the network’s structural integrity. Notably, “sleep problems,” as the principal bridging node, is important to the comorbidity of anxiety, depression, and insomnia. Addressing and preventing these key symptoms could substantially enhance the psychological well-being of the elderly, offering a promising direction for intervention strategies aimed at fostering healthier aging.

## Introduction

According to the *Statistical Yearbook* released by the National Bureau of Statistics of China (2023), the population of individuals aged 65 and older in China reached 217.09 million, representing 15.4% of the total population. This signifies that China has entered an era of significant demographic aging. As the population continues to age (Michel & Sadana, 2017), the health concerns of older adults are becoming increasingly prominent. Within the framework of healthy aging, mental health emerges as a critical indicator of well-being among the elderly. It plays a pivotal role in determining the quality of life during the later stages of life, not only influencing one’s attitudes and satisfaction but also exerting indirect effects on physical health (Cheng, Lan & Ci, 2023). Therefore, in order to achieve the goals of healthy and active aging in China, it is necessary to prioritize and address the mental health needs of the elderly population.

Sleep holds significant importance for the mental health of older adults. As the aging process advances, alterations in sleep structure are inevitable (Cohen et al., 2022). Compounded by the various health challenges that accompany aging, the prevalence of insomnia among the elderly is markedly higher compared to other age groups (Ohayon et al., 2004). An epidemiological study in the United States reported that 30%–40% of older adults experience sleep disorders (Roberts et al., 2000). A similar survey conducted in China revealed that 49.9% of elderly individuals in the country have sleep disturbances (Li et al., 2024). According to the fifth edition of the *Diagnostic and Statistical Manual of Mental Disorders* (DSM-5) (American Psychiatric Association, 2013) insomnia is considered one of the hallmark symptoms of both anxiety and depression. Epidemiological findings indicate that approximately 40% of individuals with insomnia also struggle with co-occurring mental health conditions (Ford & Kamerow, 1989). In particular, insomnia is frequently observed alongside symptoms of depression and anxiety (Ancoli-Israel, 2006). Moreover, extensive empirical research and clinical observations underscore the bidirectional relationship between sleep disturbances and the exacerbation of mental health issues such as anxiety and depression (Giannakopoulos & Kolaitis, 2021; Geng et al., 2019).

However, most previous research on anxiety, depression, and insomnia has overlooked variations in symptom importance and failed to account for the interactions between symptoms (Borsboom & Cramer, 2013; Bringmann et al., 2022). As a result, important symptom-level dynamics may have been overlooked, limiting the development of precise intervention strategies for older adults with comorbid psychological conditions. To address these gaps, the present study focuses on older adults in Jiangsu Province and examines the symptom-level associations among anxiety, depression, and insomnia. Specifically, this study aims to investigate which symptoms play key roles in node centrality and bridge strength within the anxiety–depression–insomnia symptom network among older adults, with a particular focus on cross-dimensional interactions. To address the gaps in existing research, this study focuses on older adults in Jiangsu Province to examine the network associations among anxiety, depression, and insomnia symptoms, as well as their cross-dimensional interactions.

Building on the above, the specific objectives of the study are: (1) to identify the most influential core symptoms within the anxiety–depression–insomnia network; (2) to use the clique-percolation method to detect overlapping symptoms among the anxiety, depression, and insomnia domains; (3) to determine symptoms that may serve as “bridges” across different symptom domains. This study provides both theoretical and empirical insights to guide the development of precise, targeted interventions and prevention strategies to improve the mental health of older adults.

## Participants & Method

### Participants

Participants in this study were older adults aged 65 and above from primary healthcare institutions in Jiangsu Province, China. Electronic informed consent was obtained from all participants. The survey was conducted from September 2023 to March 2024.

The inclusion criteria for older adults were: (1) aged 65 years or older; (2) absence of severe neurological or psychiatric disorders; (3) ability to actively cooperate with investigators and comprehend and respond to the questionnaire; and (4) voluntary participation. Exclusion criteria included: (1) refusal to participate; (2) communication impairments; (3) intellectual disabilities or severe psychiatric conditions; and (4) being in the terminal stage of life.

Jiangsu Province consists of 13 prefecture-level cities. For this study, three cities were selected to represent different economic levels based on their GDP rankings: Nanjing (high level), Yangzhou (medium level), and Zhenjiang (low level). From each city, two districts or counties were chosen, and within each of these administrative units, two healthcare institutions were designated as survey sites, resulting in a total of 12 survey sites. While the initial plan was to recruit approximately 230 participants per site, a total of 2,618 individuals elders completed the questionnaire across all sites.

The study was approved by the Ethics Committee of Huashan Hospital, Fudan University (Approval No. 2022-057). Recruitment involved sub-district offices, community committees, and healthcare teams *via* phone, WeChat, and flyers, targeting community and homebound elders. Participants completed an electronic questionnaire *via* scanning a Quick Response (QR) code after providing electronic informed consent. Those unable to complete it independently received face-to-face interviews with audio recording.

### Measures

The Generalized Anxiety Disorder-7 item (GAD-7) was used to measure anxiety symptoms among older adults. This instrument comprises seven items rated on a 4-point Likert scale, measuring anxiety in the past years. Each item is scored from 0 to 3 (“not at all”, “several days”, “more than half the days”, “almost every day”), yielding a total score ranging from 0 to 21. Higher scores indicate greater severity of anxiety. Based on the total score, anxiety severity is classified as follows: 0–4 (no anxiety), 5–9 (mild anxiety), 10–14 (moderate anxiety), and ≥15 (severe anxiety) (Spitzer et al., 2006). Participants with GAD-7 scores ≥5 were categorized as exhibiting symptoms of anxiety severity (*i.e.,* mild, moderate and severe anxiety symptoms), hereafter referred to as ‘anxiety symptoms’ for simplicity.

The Patient Health Questionnaire-9 (PHQ-9) is a widely used, self-administered screening and assessment instrument for depression. It consists of nine items, each corresponding to one of the diagnostic criteria for major depressive disorder as defined in the DSM-IV. Each item is rated on a 4-point scale from “0” (not at all) to “3” (nearly every day), with higher scores indicating more greater severity of depressive symptoms. Depression severity is categorized as follows: 0–4 (none), 5–9 (mild), 10–14 (moderate), 15–19 (moderately severe), and 20–27 (severe) (Kroenke, Spitzer & Williams, 2001). Scores of 5 or above were classified as indicating depressive severity symptoms (*i.e.,* mild to severe depressive symptoms), hereafter referred to simply as ‘depressive symptoms’.

The severity of insomnia was assessed using the Insomnia Severity Index (ISI), a self-reported instrument in which each item is rated on a 5-point Likert scale ranging from 0 to 4. The ISI evaluates insomnia symptoms over the past month across seven domains: difficulty falling asleep, staying asleep, early morning awakenings, dissatisfaction with sleep, the impact of sleep problems on daytime functioning, the extent to which others notice these problems, and the level of distress they cause. Total scores range from 0 to 28 and are interpreted as follows: 0–7 (no insomnia), 8–14 (subthreshold insomnia), 15–21 (moderate insomnia), and 22–28 (severe insomnia) (Morin et al., 2011). Participants scoring 8 or above were considered to exhibit insomnia symptoms, hereafter abbreviated as ‘insomnia symptoms’.

### Data analysis

The descriptive statistical analysis and network analysis were conducted using R software (version 4.4.1; R Core Team, 2024).

#### Missing value processing

The demographic data exhibited the following patterns of missingness: 26 cases were absent for marital status, and 152 cases were missing for monthly household income. All other demographic variables were complete. To address the missing data, the K-nearest neighbors (KNN) imputation method was employed. As completion of the GAD-7 and the PHQ-9 were mandatory, no missing data were observed for these measures. However, 532 participants had missing data on ISI and were excluded from the analysis, resulting in a final analytic sample size of 2,086 participants.

#### Network estimation

The R package “qgraph” was utilized to construct a symptom network diagram employing the EBICglasso function alongside Spearman’s correlation analysis (Epskamp, Borsboom & Fried, 2018). In this network model, each symptom is represented as a node, with the associations between nodes depicted as edges. However, when the number of nodes is large, displaying all possible connections can result in an overly dense and difficult-to-interpret network. To enhance interpretability and reduce clutter, the Least Absolute Shrinkage and Selection Operator (LASSO) and the Bayesian Information Criterion (EBIC) were applied to shrink the edge weight and optimize the selection of tuning parameters (Epskamp, Borsboom & Fried, 2018).

Furthermore, the “mgm” package in R was used to assess the predictability of all nodes in the network, as it supports mixed data types, including both continuous and categorical variables. Nodes with higher predictability values suggest that their fluctuations can largely be accounted for by changes in neighboring nodes. The average predictability across all nodes serves as an indicator of the internal consistency and explanatory power of the network as a whole. A higher average predictability implies that the network structure is more adept at internally predicting the behavior of its components, with external factors contributing less to the observed variation (Liu et al., 2022).

#### Cluster identification

To identify meaningful symptom clusters within the network, overlapping communities were examined using the Clique Percolation Method (CPM) (Derényi, Palla & Vicsek, 2005). CPM conceptualizes communities as groups of fully connected subgraphs (cliques) that share a subset of their nodes. The algorithm first detects all k-cliques in the estimated network and subsequently constructs a secondary graph in which each k-clique is treated as a node. Edges are added between two clique-nodes if they share k–1 nodes, and each connected component in this secondary graph is defined as a community. In the present study, *k* = 5 was selected to detect percolated nodes, allowing for the identification of symptoms that simultaneously belong to multiple communities. Visualization of these overlapping communities was achieved using the “cpColoredGraph” function in the CliquePercolation package (Lee, Cao & Gonzalez-Guarda, 2023).

#### Network centrality

In network analysis, three centrality metrics are commonly employed to assess the prominence of nodes: strength centrality, betweenness centrality, and closeness centrality. Previous research has indicated that the stability of betweenness and closeness centrality tends to be relatively low (Birkeland, Greene & Spiller, 2020). Therefore, when discrepancies arise among the ranking produced by these measures, strength centrality is typically considered as the most reliable indicator. In this study, R “qgraph” package was used to compute strength centrality, defined as the sum of the absolute weights of the edges connecting a given node to other nodes. Nodes with higher strength centrality are more strongly connected to other nodes, signifying their greater importance within the network. In the context of a symptom network, such nodes are generally regarded as the key symptoms driving the structure of the network.

Moreover, we examined the interconnections between groups of nodes representing different variables, identifying those nodes that serve as key connectors between two distinct groups, referred to as bridge nodes. The strength of these bridge nodes was quantified using the R package “networktools”. A higher bridge strength indicates a more pivotal role for that node in facilitating or maintaining connections across different groups or sub-networks. In other words, the greater strength of a bridge node, the more integral it is in fostering the interactions between disparate clusters. Based on the standardized bridge strength within the network, the top 20% of nodes are selected as the predicted bridge nodes for this study (Birkeland, Greene & Spiller, 2020).

#### Network stability

We used the R package “bootnet” to assess the accuracy and stability of the network model. The correlation stability coefficient (CS-C) was computed using the case-dropping subset bootstrap method to evaluate the stability of centrality indices. A CS-C value greater than 0.25 is generally considered as moderate stability, whereas a value exceeding 0.5 suggests high stability (Epskamp, Borsboom & Fried, 2018). To examine the robustness of node strength and bridge strength, non-parametric bootstrapping was conducted 1,000 times to assess any significant differences.

## Results

### Demographic characteristics

A total of 2,086 older adults were included in this study. The mean age was 72.5 years (standard deviation (SD) = 5.0), 1,025 (50.9%) were male. Detailed demographic characteristics of the participants are shown in Table 1.

**Table 1:** Demographic characteristics of the elderly population.

**Variable**		**Summary**
Age, mean (sd)		72.49 (4.95)
BMI, mean (sd) (kg/m^2^)		24.4 (3.6)
Sex, n (%)	Male	1,025 (49.14%)
	Female	1,061 (50.86%)
Marital status, n (%)	Married	1,716 (82.26%)
	Unmarried	26 (1.25%)
	Widowed	325 (15.58%)
	Divorced	19 (0.91%)
Educational attainment, n (%)	Primary and below	1,266 (60.69%)
	Junior/Secondary	544 (26.08%)
	High school and above	276 (13.23%)
Current place of residence, n (%)	Rural	1,143 (54.79%)
	Urban	943 (45.21%)
Monthly household income, n (%)	<3,000	706 (33.84%)
	3,000–6,000	660 (31.64%)
	>6,000	720 (34.52%)
Cigarette smoking, n (%)	Never smoked	1,676 (80.35%)
	Tobacco cessation	75 (3.60%)
	Cigarette smoking	335 (16.05%)
Drinking wine, n (%)	Never drink alcohol	1,792 (85.90%)
	A ban on alcohol	46 (2.21%)
	Drinking wine	248 (11.89%)
Live alone, n (%)	No	1,850 (88.69%)
	Yes	236 (11.31%)
Satisfaction with housing and surroundings environment, n (%)	No	220 (10.55%)
	Yes	1,866 (89.45%)
Interest in hobbies, n (%)	No	1,104 (52.92%)
	Yes	982 (47.08%)
Participation of social activities, n (%)	No	1,481 (71.00%)
	Yes	605 (29.00%)
Nutritional status, n (%)	Malnourished	932 (44.68%)
	Normal nutrition	1,154 (55.32%)

### Descriptive statistics of the GAD-7, PHQ-9, and ISI-7 scales

Among the 2,086 participants, the internal consistency of the scales was high, with Cronbach’s α coefficients of 0.865 for the GAD-7, 0.882 for the PHQ-9, and 0.950 for the ISI. The mean total scores for the PHQ-9, GAD-7, and ISI were 2.5 (standard deviation (SD) = 3.4), 1.5 (SD = 2.5), and 9.5 (SD = 5.8), respectively. Median scores and interquartile ranges (IQR) were 0 (0, 2) for the GAD-7, 1 (0, 4) for the PHQ-9, and 6 (5, 12) for the ISI.

**Figure 1:**
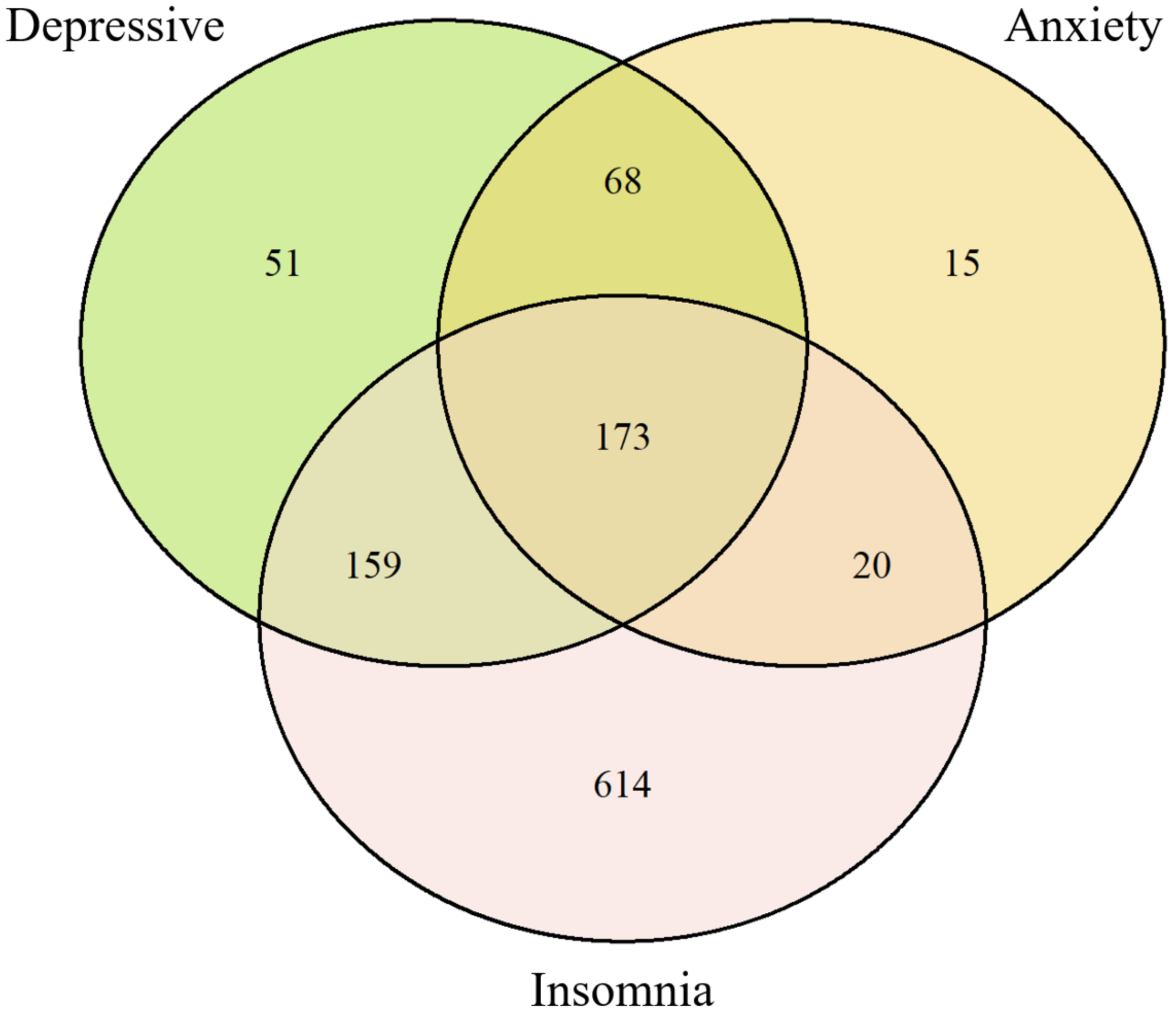
Venn diagram illustrating the overlap among anxiety, depressive, and insomnia.

Anxiety severity measured by the GAD-7 was categorized as no anxiety in 1,810 individuals (86.8%), mild anxiety in 251 (12.0%), moderate anxiety in 22 (1.2%), and severe anxiety in 3 (0.1%). According to the PHQ-9, 1,635 individuals (78.4%) had no depression, 376 (18.0%) had mild depression, 53 (2.5%) moderate, 17 (0.8%) moderately severe, and 5 (0.2%) severe depression. Based on the ISI-7, 1,120 participants (53.7%) reported no insomnia, 612 (29.3%) had subthreshold insomnia, 254 (12.2%) moderate insomnia, and 78 (3.7%) severe insomnia. The prevalences of anxiety (GAD-7 score ≥ 5), depressive (PHQ-9 score ≥ 5), and insomnia (ISI score ≥ 8) were 21.6% (95% confidence interval (CI) [19.9%–23.4%]), 13.2% (95% CI [11.8%–14.7%]), and 46.3% (95% CI [44.2%–48.5%]), respectively. The Venn diagram (Fig. 1) displays the overlap among participants reporting symptoms of depression, anxiety, and insomnia. A total of 1,100 participants with at least one symptom, 173 individuals reported all three symptoms concurrently.

### Network analysis

#### Network structure

**Figure 2:**
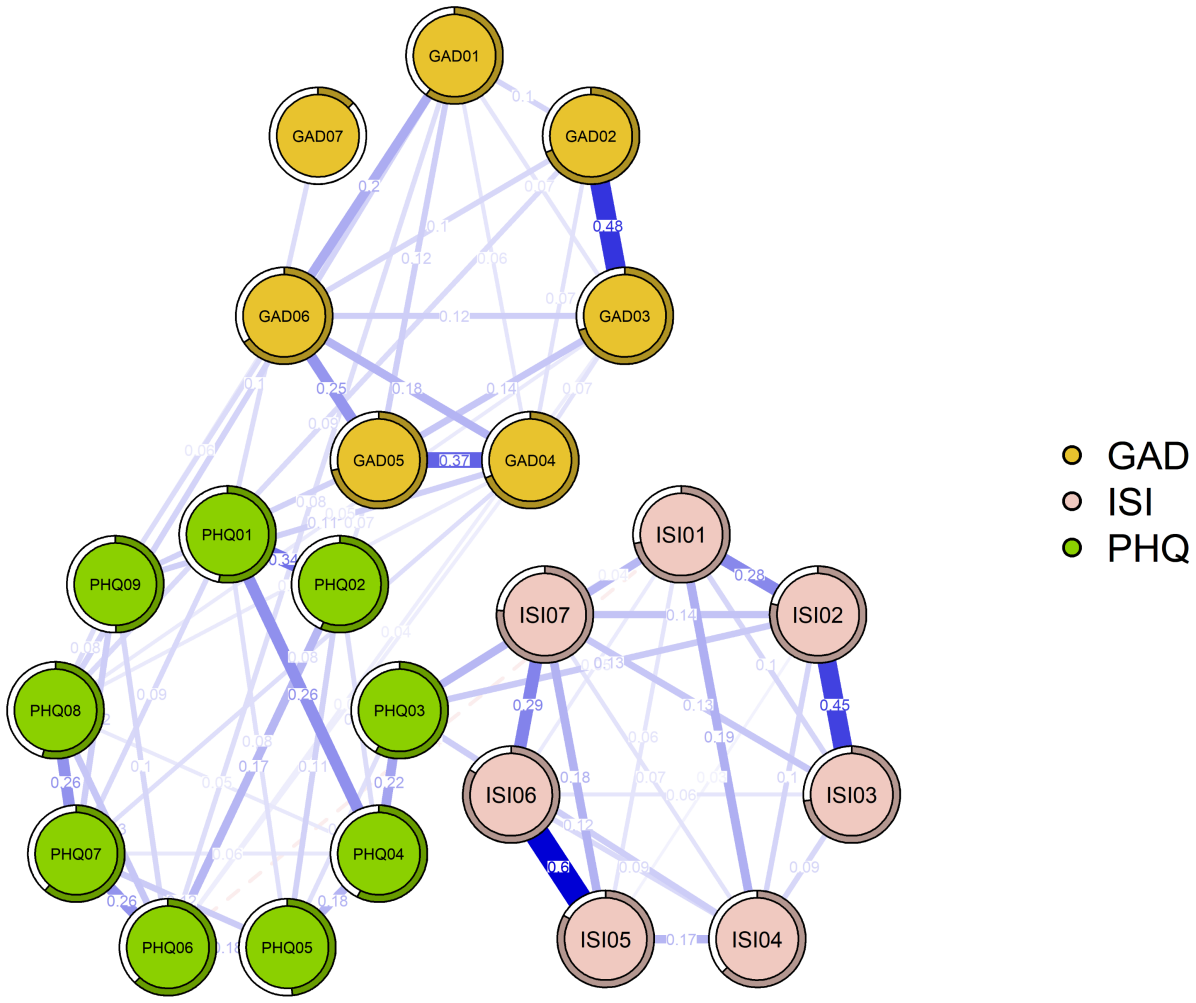
Network structure ofanxiety, depression and insomnia in the elderly.

Figure 2 shows the network structure of anxiety, depression, and insomnia. The network illustrates a closely association between anxiety and depression, while insomnia appears relatively isolated, with fewer connections to the symptoms of anxiety and depression. Among the 190 edges, 71 (37.4%) exhibited non-zero regularized partial correlation coefficients. The network model and edge weight matrix reveal that the strongest positive edges within their respective communities are between “Noticeability of sleep problems by others” and “Distress caused by sleep difficulties” (ISI05–ISI06, edge weight = 0.60), “Uncontrollable worry” and “Excessive worry” (GAD02–GAD03, edge weight = 0.48), and “Severity of sleep onset” and “Early morning waking problems” (ISI01–ISI03, edge weight = 0.45). The only negative edge in the network is between “Guilt” and “Severity of sleep onset” (PHQ06–ISI01, edge weight = −0.04). For the regularized partial correlation coefficients of each edge, refer to Fig. S1.

Figure 2 illustrates the network structure of anxiety, depression, and insomnia symptoms among older adults in Jiangsu. Each node represents a specific symptom item from one of three standardized instruments: the GAD-7 for anxiety (orange nodes), the PHQ-9 for depression (green nodes), and the ISI-7 for insomnia (pink nodes). The layout is organized by symptom domains, showing clear clustering within each domain.

The edges represent the regularized partial correlation coefficients between two nodes. Solid blue lines indicate positive correlations, whereas dashed red lines represent negative correlations. Thicker edges correspond to stronger associations. Each pie chart around the node indicates the predictability of such node, as the proportion of its variance explained by all other nodes in the network. Categories such as ISI5 (“dissatisfaction with current sleep”) and ISI6 (“distress caused by sleep problems”) have the thickest connecting edge and high predictability, suggesting a strong reciprocal influence between these two symptoms.

#### Clique percolation cluster identification

**Figure 3:**
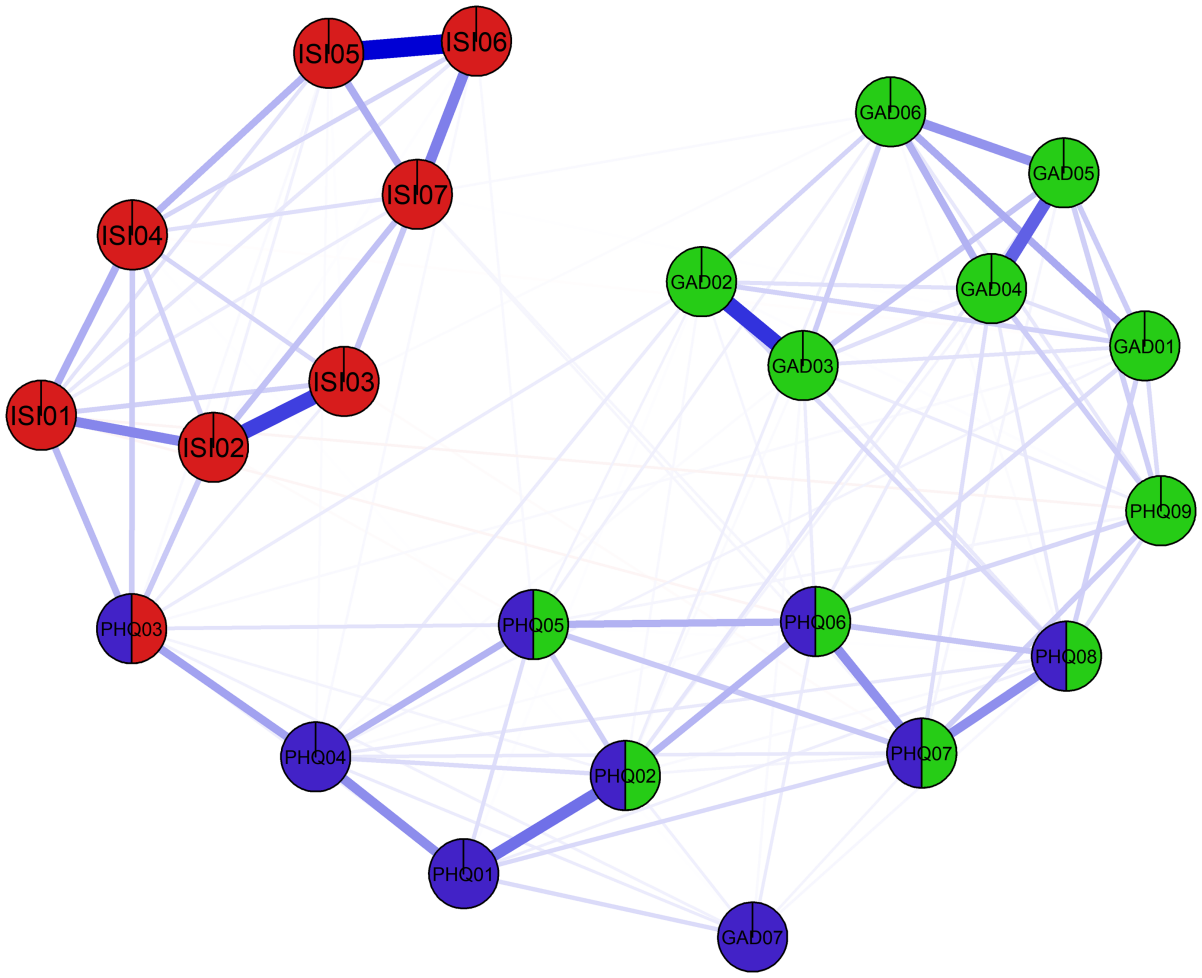
Clique percolation network containing items from anxiety, depression, and insomnia.

Inspection of the Clique Percolation plot identified three clusters as outline below. This plot is displayed in Fig. 3.

 The first cluster included all ISI nodes and also included PHQ03 (Sleep problems). The second cluster consisted of GAD01 through GAD06 nodes along with PHQ02, PHQ05, PHQ06, PHQ08, and PHQ09 nodes. The third cluster included PHQ01 through PHQ08 nodes together with GAD07.

Among these clusters, PHQ03 (Sleep problems) was a percolated node overlapping with the insomnia cluster, while PHQ02 (Sad Mood), PHQ05 (Appetite), PHQ06 (Guilt), PHQ07 (Concentration), and PHQ08 (Motor problems) overlapped with the anxiety cluster. PHQ09 (Suicide ideation) was fully assigned to the anxiety cluster, and GAD07 (Fear something awful; Feeling afraid) was fully assigned to the depression cluster.

#### Key nodes and bridge nodes

**Figure 4:**
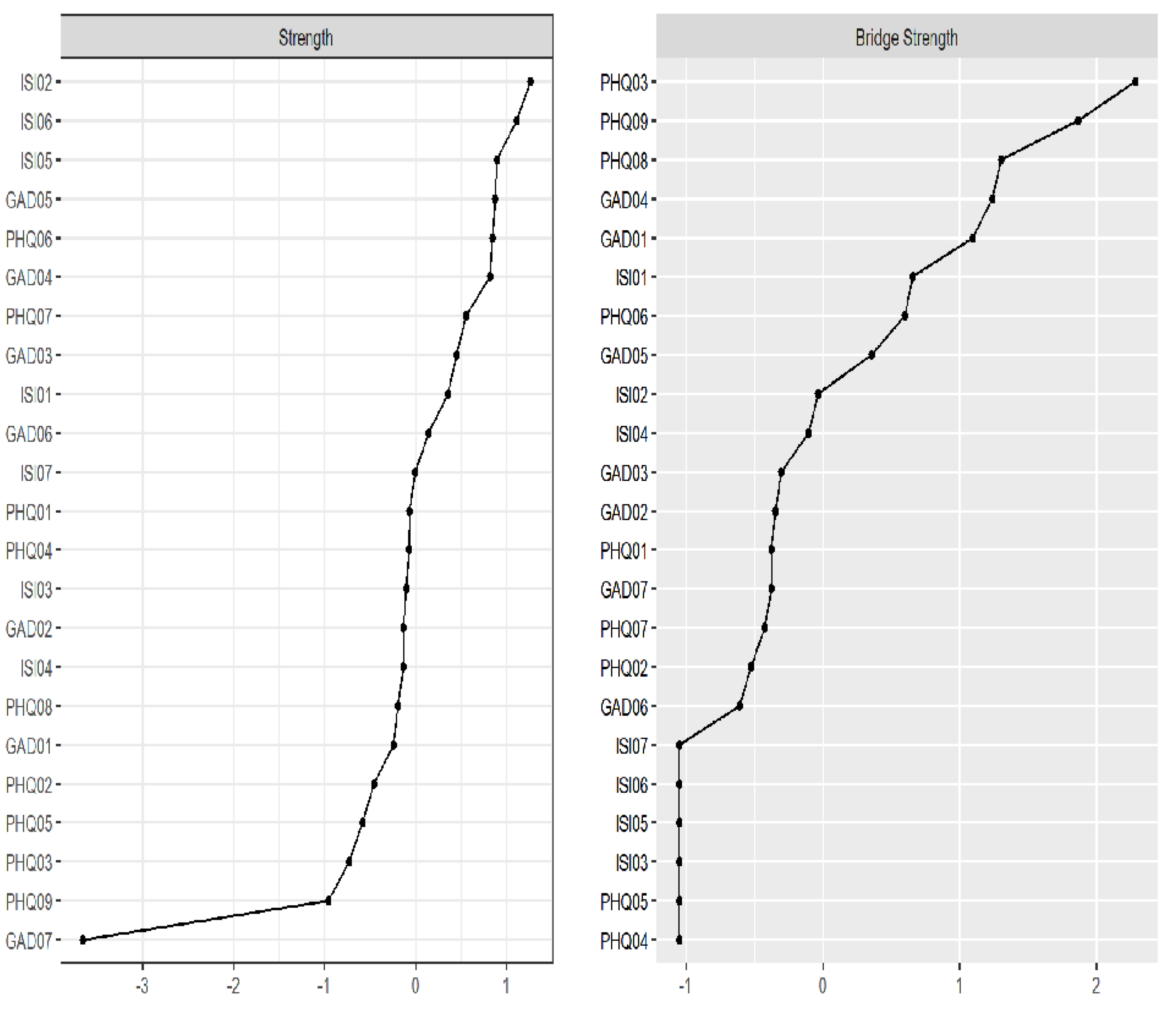
Strength and bridge strength of symptoms.

Figure 4A illustrates the strength centrality of symptoms within the network. “Sleep maintenance” (ISI02, strength = 1.134) is the most central node, followed by “Distress caused by sleep difficulties” (ISI06, strength = 1.101), “Noticeability of sleep problems by others” (ISI05, strength = 1.055) and “Restlessness” (GAD05, strength = 1.051), all of which demonstrated statistically higher strength values compared to most other nodes in the network (Fig. S2). Conversely, “Feeling afraid” (GAD07, strength = 0.084) is the least central node in the network.

Figure 4B shows the bridge strength of the network. “Sleep problems” (PHQ03, bridge strength = 0.416), “Suicide ideation” (PHQ09, bridge strength = 0.364), “Motor problems” (PHQ08, bridge strength = 0.294) and “Trouble relaxing” (GAD04, bridge strength = 0.285) are the strongest bridging symptoms connecting anxiety, depression and insomnia. Additional centrality indices are provided in Fig. S3. The specific values of each item are shown in Table 2.

**Table 2:** Descriptive statistics for PHQ-9, GAD-7 and ISI-7 entries.

**Acronyms**	**Content of entries**	**Mean (SD)**	**Dissociation**	**Bridge strength**	**Predictability**
GAD1	Nervousness	0.20 (0.45)	0.812	0.268	0.600
GAD2	Uncontrollable worry	0.23 (0.48)	0.836	0.088	0.694
GAD3	Excessive worry	0.20 (0.46)	0.960	0.093	0.701
GAD4	Trouble relaxing	0.16 (0.42)	1.039	0.285	0.690
GAD5	Restlessness	0.14 (0.39)	1.051	0.176	0.716
GAD6	Irritability	0.18 (0.42)	0.894	0.055	0.657
GAD7	Fear something awful Feeling afraid	0.36 (0.71)	0.084	0.084	0.131
PHQ1	Anhedonia	0.36 (0.58)	0.850	0.084	0.530
PHQ2	Sad Mood	0.25 (0.50)	0.766	0.066	0.565
PHQ3	Sleep problems	0.56 (0.80)	0.708	0.416	0.584
PHQ4	Fatigue	0.41 (0.60)	0.849	0.000	0.574
PHQ5	Appetite	0.24 (0.47)	0.740	0.000	0.483
PHQ6	Guilt	0.19 (0.45)	1.044	0.206	0.619
PHQ7	Concentration	0.20 (0.47)	0.983	0.078	0.612
PHQ8	Motor problems	0.22 (0.48)	0.823	0.294	0.545
PHQ9	Suicide ideation	0.09 (0.34)	0.660	0.364	0.498
ISI1	Severity of sleep onset	0.69 (0.95)	0.940	0.213	0.720
ISI2	Sleep maintenance	1.57 (0.90)	1.134	0.127	0.786
ISI3	Early morning wakening problems	1.57 (0.91)	0.842	0.000	0.729
ISI4	Sleep dissatisfaction	1.84 (1.12)	0.836	0.118	0.629
ISI5	Noticeability of sleep problems by others	1.65 (0.95)	1.055	0.000	0.831
ISI6	Distress caused by sleep difficulties	1.61 (0.90)	1.101	0.000	0.836
ISI7	Interference with daytime functioning	0.54 (0.86)	0.863	0.000	0.764

#### Network stability

**Figure 5:**
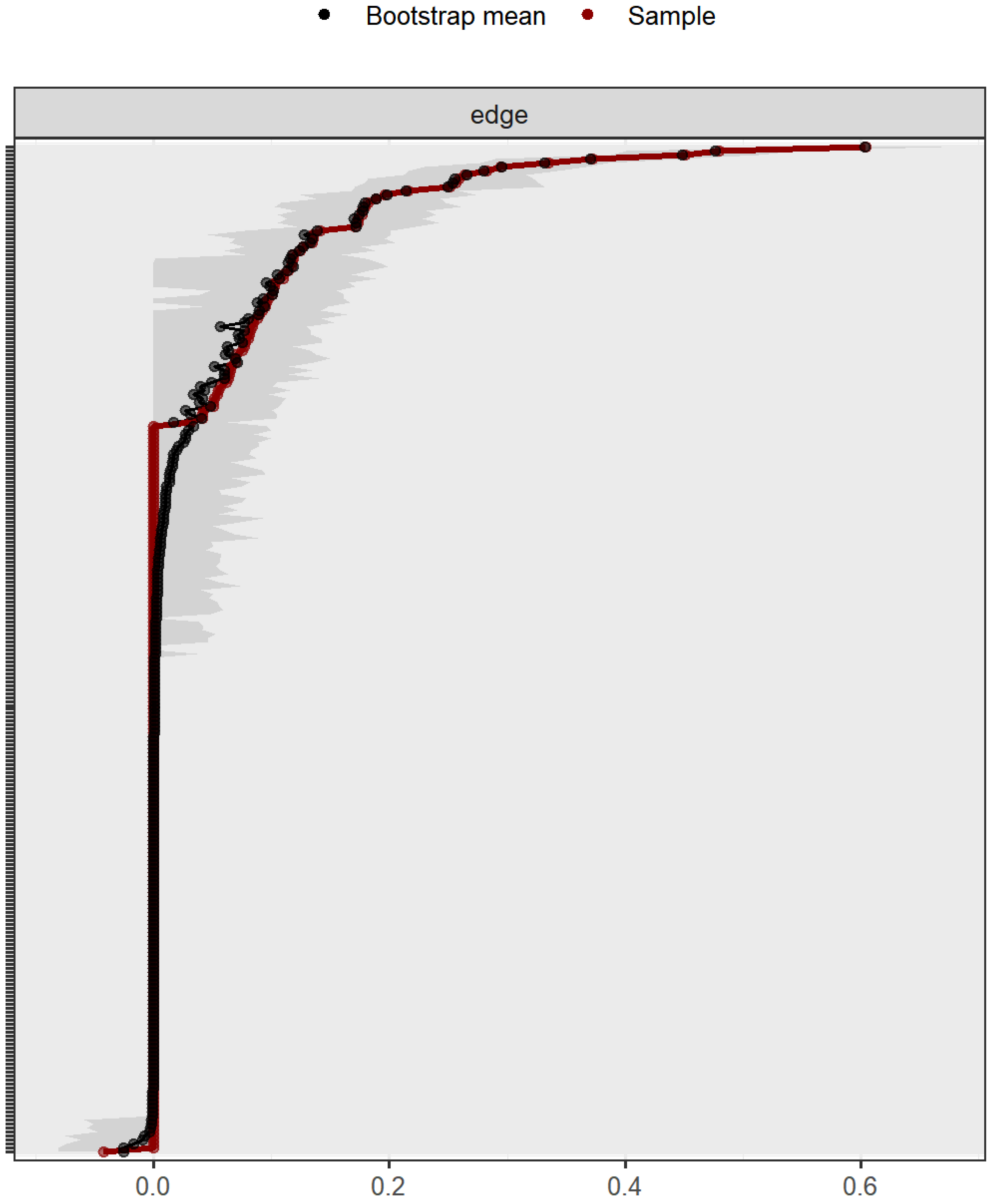
Accuracy of edge weights in network models.

The bootstrap method was used to assess the stability of the network structure. In Fig. 5, red dots represent the edge weights derived from the original dataset, while black dots denote the bootstrap mean of these edge weights, arranged in descending order. The close alignment between the original sample values and their bootstrap mean indicates that the estimated edge weights are highly stable. The gray shading represents the 95% confidence intervals (CIs) of the edge weights, estimated using the non-parametric bootstrap method. A narrower CI indicates higher stability. Figure 5 demonstrates that the relatively narrow 95% CIs suggest high precision and low uncertainty in the estimated network edges, with most CIs of the edge weights overlapping to some extent.

**Figure 6:**
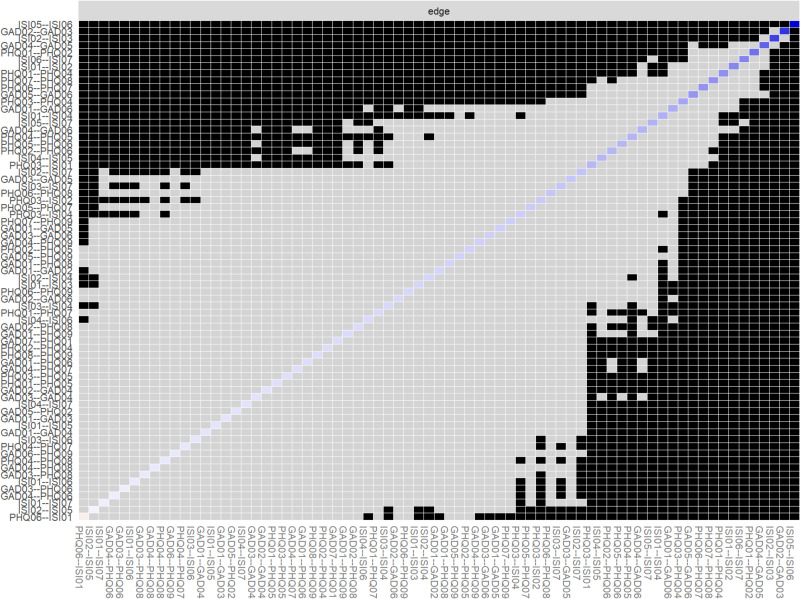
Difference in edge weights estimated by bootstrap variance estimation.

In Fig. 6, grey boxes indicate edges with no significant difference in edge weight, and black boxes indicate edges with significant differences in edge weight (*α* = 0.05). Blue boxes in the edge weight plot indicate positive correlations, and orange boxes indicate negative correlations. The estimation of the difference in edge weights shows that the edges with higher stability are significantly different from the other edges in the network.

**Figure 7:**
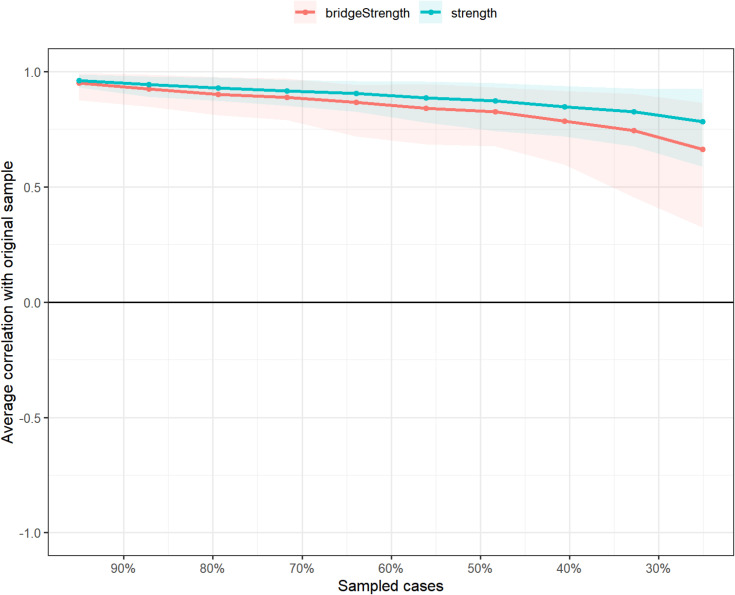
The stability of strength and bridge strength indices using case-dropping bootstrap.

Figure 7 illustrates the average correlation of network metrics (bridge strength and strength) with the original sample, as a function of the proportion of sampled cases. The *x*-axis indicates the percentage of the original sample cases, and the *y*-axis indicates the average correlation between the centrality measures obtained from the original network and the network re-estimated networks. The blue and red lines correspond to bridge strength and strength, respectively, with shaded regions showing the 95% CIs. The decreasing trend suggests that as fewer cases are sampled, the correlation with the original network decreases, with bridge strength showing slightly lower stability compared to strength. At the same time, the analysis of the node strength value and the bridge strength value shows that their CS coefficients are 0.75 and 0.594, respectively, both falling within an acceptable range, thereby supporting the reliability of these centrality metrics.

## Discussion

Based on the psychopathology network theory proposed by Borsboom (2017) and McNally (2016), this study is the first to employ network analysis to investigate the comorbidity among anxiety, depression, and insomnia symptoms in the elderly population. By evaluating symptom–symptom associations through edge weights, the core pathways underlying the development of mental disorders can be revealed. In this network, we observed that the strongest connections are predominantly confined to a single cluster of nodes, a finding that aligns with previous research (Liu et al., 2024; Groen et al., 2020). Notably, the most robust connection in our network is between ISI5 and ISI6 (“Noticeability of sleep problems by others” and “Distress caused by sleep difficulties”), which contrasts with findings from a study conducted during the COVID-19 pandemic among Macau residents, where the most significant association was observed between ISI1 and ISI2 (“Severity of sleep onset” and “Sleep maintenance”) (Bai et al., 2022). This discrepancy may be attributed to differences in the study populations and temporal contexts. Specifically, the participants in our study were elderly individuals, and prior research has demonstrated that the risk of reduced daily functioning escalates with age (Chatterji et al., 2015). Older adults are often more reliant on familial or caregiving support, and their health status is typically a central concern for their families. Consequently, the physical and psychological changes they experience are more readily observed by those around them, potentially explaining the stronger link between ISI5-ISI6 (“Noticeability of sleep problems by others” and “Distress caused by sleep difficulties”). In this context, ISI06 (“Distress caused by sleep difficulties”) exhibited the highest predictability in the network (*R*^2^ = 0.836), primarily due to its central position within the insomnia symptom network and its strong associations with multiple insomnia symptoms. Specifically, ISI06 showed the strongest correlation with ISI05 (“Noticeability of sleep problems by others”, *r* = 0.60) and a moderate correlation with ISI07 (“Interference with daytime functioning”, *r* = 0.27). These strong inter-node connections indicate that variations in ISI06 can be highly explained by the states of its neighboring nodes, resulting in its highest predictability. Furthermore, the network revealed that many of the strongest associations occur between insomnia symptoms, underscoring the prominence of sleep disturbances within the elderly population in Jiangsu Province. Given these findings, it is clear that addressing sleep-related issues should be prioritized in the development of targeted interventions and health measures.

Based on clique percolation analysis, we further identified key symptoms that play cross-dimensional roles among insomnia, anxiety, and depression. Specifically, PHQ03 (“Sleep problems”) was found to belong to both the insomnia and depression clusters, reinforcing the notion that sleep problems have cross-diagnostic characteristics and serve as a critical mechanism for the occurrence and maintenance of depression. Additionally, PHQ02 (“Sad Mood”), PHQ05 (“Appetite”), PHQ06 (“Guilt”), PHQ07 (“Concentration”), and PHQ08 (“Motor problems”) overlapped with the anxiety cluster, indicating that depressive emotional and functional impairments often co-occur with anxious emotional activation and cognitive load. This pattern is consistent with previous evidence of high comorbidity between anxiety and depression (Groen et al., 2020; Owczarek et al., 2022). Notably, PHQ09 (“Suicidal ideation”) was fully assigned to the anxiety cluster, whereas GAD07 (“Fear something awful”) was fully assigned to the depression cluster. The former may relate to heightened threat perception and emotion regulation difficulties in anxious individuals, while the latter reflects the core role of catastrophizing in depression. These findings suggest that traditional boundaries between anxiety and depression are blurred within the symptom network, and clique percolation analysis can effectively capture such cross-dimensional coupling structures (Ribeiro Santiago et al., 2024).

Node strength serves as an important indicator for identifying core symptoms within symptom networks (Hevey, 2018). These nodes not only activate other nodes but also play a key role in driving interactions within the network, thereby exerting a significant influence on the overall network structure. In this study, ISI2 (“Sleep maintenance”) exhibited the highest node strength within the insomnia symptom network, suggesting its pivotal role in maintaining the overall structure of the network and highlighting its potential as a target for interventions aimed at alleviating other symptoms within the anxiety-depression-insomnia triad (McElroy & Patalay, 2019). This finding contrasts with prior research (Dekker, Blanken & Van Someren, 2017), which identified ISI4 (“Sleep dissatisfaction”) as the most influential symptom within the intersection of insomnia and personality traits, reflecting the specificity of symptom networks in older adults. This distinction may be explained by the unique nature of aging, which induces notable alterations in sleep architecture—older adults experience reductions in deep sleep and Rapid Eye Movement (REM) sleep, along with diminished sleep efficiency and prolonged sleep latency compared to younger individuals (Ohayon et al., 2004). Furthermore, older adults often have multiple chronic conditions, and both the diseases themselves and the medications used to manage them can exacerbate sleep disturbances. Pain and discomfort, for example, can cause nocturnal awakenings, while certain medications may disrupt sleep further or alter sleep patterns (Brewster, Riegel & Gehrman, 2022). As a result, older adults frequently experience fragmented sleep and difficulty returning to sleep. Cognitive behavioral therapy for insomnia (CBT-I) has been shown to be particularly effective in alleviating persistent insomnia and its associated symptoms, such as anxiety and fatigue, in elderly populations with comorbidities (Lovato, Lack & Kennaway, 2016; Mitchell et al., 2019). Additionally, pharmacological treatments, including sedative-hypnotics, can provide rapid relief for sleep disturbances, with benzodiazepines (Schroeck et al., 2016) and non-benzodiazepines (Smagula et al., 2016) demonstrating efficacy in improving sleep in individuals with primary insomnia.

The other two key nodes are ISI6 (“Distress caused by sleep difficulties”) and ISI5 (“Noticeability of sleep problems by others”) among insomnia symptoms, which, similar to node ISI2, largely reflect the impact of aging on sleep quality. All the key symptoms identified have a high degree of predictability, accounting for 63.0% of the variance in the average predictability of all nodes in the network. This indicates that 63.0% of the variation in the overall network structure can be maintained and explained by the network itself, meaning that the variance of most nodes can be well explained by their adjacent nodes. This suggests that the network structure is clear, the relationships between nodes are strong and close, and interventions targeting the key nodes may have a significant impact on the overall network.

The identification of bridge symptoms among different psychopathological symptoms is equally important, as these nodes can facilitate the spread of comorbidity and hold potential for intervention research (Contreras et al., 2019; Byrne et al., 2021; Haws et al., 2022). These symptoms should be given particular attention in treatment research to reduce the risk of interaction between mental syndromes (Jones, Ma & McNally, 2021). In the insomnia-anxiety-depression symptom network, the depressive symptom PHQ3 (“Sleep problems”) is the node with the strongest bridging strength. “Sleep problems” refer to difficulty falling or staying asleep, or the distress of sleeping too much (Kroenke, Spitzer & Williams, 2001). As shown in Supplementary 1, there is a strong correlation between this symptom and another insomnia symptom, ISI1 (“Severity of sleep onset”), suggesting that in addition to maintenance insomnia, primary insomnia may also be a key symptom that sustains the entire network and should be an important target for intervention. Some studies (Zheng et al., 2020) have shown that certain drugs (such as Triazolam, Zopiclone, Zaleplon, and Remeron) can effectively improve the symptoms of difficulty falling asleep. Additionally, a meta-analysis (Cheung et al., 2019) indicated that psychological interventions can also improve sleep latency and total sleep time. The three nodes with the strongest bridge strength are all related to depressive symptoms: PHQ3 “Sleep problems”, PHQ9 “Suicidal ideation”, and PHQ8 “Motor problems”. This suggests that interventions targeting these three depressive symptoms not only improve depressive symptoms but also have a conditional and controlling effect on the two interacting node groups.

Finally, despite the valuable findings and insights of this study, there are several limitations. First, this study is based on cross-sectional data, which makes it impossible to establish a definitive causal relationship between symptoms. Therefore, future research should collect longitudinal data and analyze their dynamic relationships to provide more robust and rigorous results that can guide clinical practice. Second, the three scales used in this study are all self-report measures, which may be subject to recall bias and social desirability effects. Third, the elderly participants in this study were all volunteers. Although the sample size was sufficient to meet statistical analysis requirements, it was not a large-scale sample, which may introduce selection bias. As such, caution is warranted when generalizing the findings of this study.

Despite the limitations mentioned above, the current study has several notable strengths. First, it is the first network analysis to explore the relationships between anxiety, depression, and insomnia symptoms in the elderly, providing insights into the structural characteristics of the symptom network in this population. The three most central symptoms in the network are ISI02 (“Sleep maintenance”), ISI06 (“Distress caused by sleep difficulties”), and ISI05 (“Noticeability of sleep problems by others”). Additionally, the bridge nodes in the overall anxiety-depression-insomnia network include PHQ03 (“Sleep problems”), PHQ09 (“Suicidal ideation”), and PHQ08 (“Motor problems”). PHQ3 acts as a bridge node in the network, exhibiting the highest bridging strength. Although it belongs to the depression cluster, it is strongly associated with insomnia symptoms (such as ISI1 “Severity of sleep onset”, ISI2 “Sleep maintenance”, and ISI6 “Distress caused by sleep difficulties”), reflecting its cross-dimensional nature. Clique percolation analysis further indicates that PHQ3 connects multiple communities of depression and insomnia, enabling it to serve as a “bridge” within the symptom network and facilitate interactions between different symptom clusters. This suggests that interventions targeting PHQ3 may not only alleviate depressive symptoms but also exert cascading effects on other related symptoms by modulating the insomnia symptom cluster.

In conclusion, the results of this study in the elderly population of Jiangsu Province indicate that insomnia-related symptoms hold both high node centrality and bridge centrality in the anxiety–depression–insomnia network, making them the most critical symptoms within the network. This suggests that difficulties in sleep maintenance, distress caused by sleep problems, and sleep issues noticeable to others occupy a central role in older adults’ mental health and may act as “bridges” connecting anxiety, depression, and insomnia symptoms. Targeted interventions addressing these core and bridge symptoms could more effectively alleviate anxiety and depressive symptoms and reduce the risk of comorbid psychological disorders among the elderly. Furthermore, these findings provide a theoretical basis for developing precise, population-specific mental health interventions, contributing to improved psychological well-being, enhanced quality of life for older adults, and offering practical guidance for public health strategies and healthy aging policies.

##  Supplemental Information

10.7717/peerj.20868/supp-1 Supplemental Information 1 Insomnia Severity Index (ISI) questionnaire administered to the elderly population in Jiangsu Province

10.7717/peerj.20868/supp-2 Supplemental Information 2 Generalized Anxiety Disorder-7 (GAD-7) and Patient Health Questionnaire-9 (PHQ-9) scales administered to the elderly population in Jiangsu Province

10.7717/peerj.20868/supp-3 Supplemental Information 3 Network Analysis code

10.7717/peerj.20868/supp-4 Supplemental Information 4 STROBE Checklist

10.7717/peerj.20868/supp-5 Supplemental Information 5 Correlation matrix of GAD-7 , PHQ-9 and ISI-7 entry scores

10.7717/peerj.20868/supp-6 Supplemental Information 6 Sociodemographic characteristics

10.7717/peerj.20868/supp-7 Supplemental Information 7 Estimation of node strength differences by bootstrap difference test

10.7717/peerj.20868/supp-8 Supplemental Information 8 CMP code

10.7717/peerj.20868/supp-9 Supplemental Information 9 Estimated network node centrality and bridge centrality

10.7717/peerj.20868/supp-10 Supplemental Information 10 Translation Codebook

## Additional Information and Declarations

### Data Availability

The following information was supplied regarding data availability: The data and code are available in the Supplemental Files.
