# Supplementary figures and images for: Network analysis of anxiety, depression and insomnia in the elderly in Jiangsu Province
Source: PeerJ. 2026 Mar 31;14:e20868. doi: 10.7717/peerj.20868 (PMC13048223; doi:10.7717/peerj.20868)

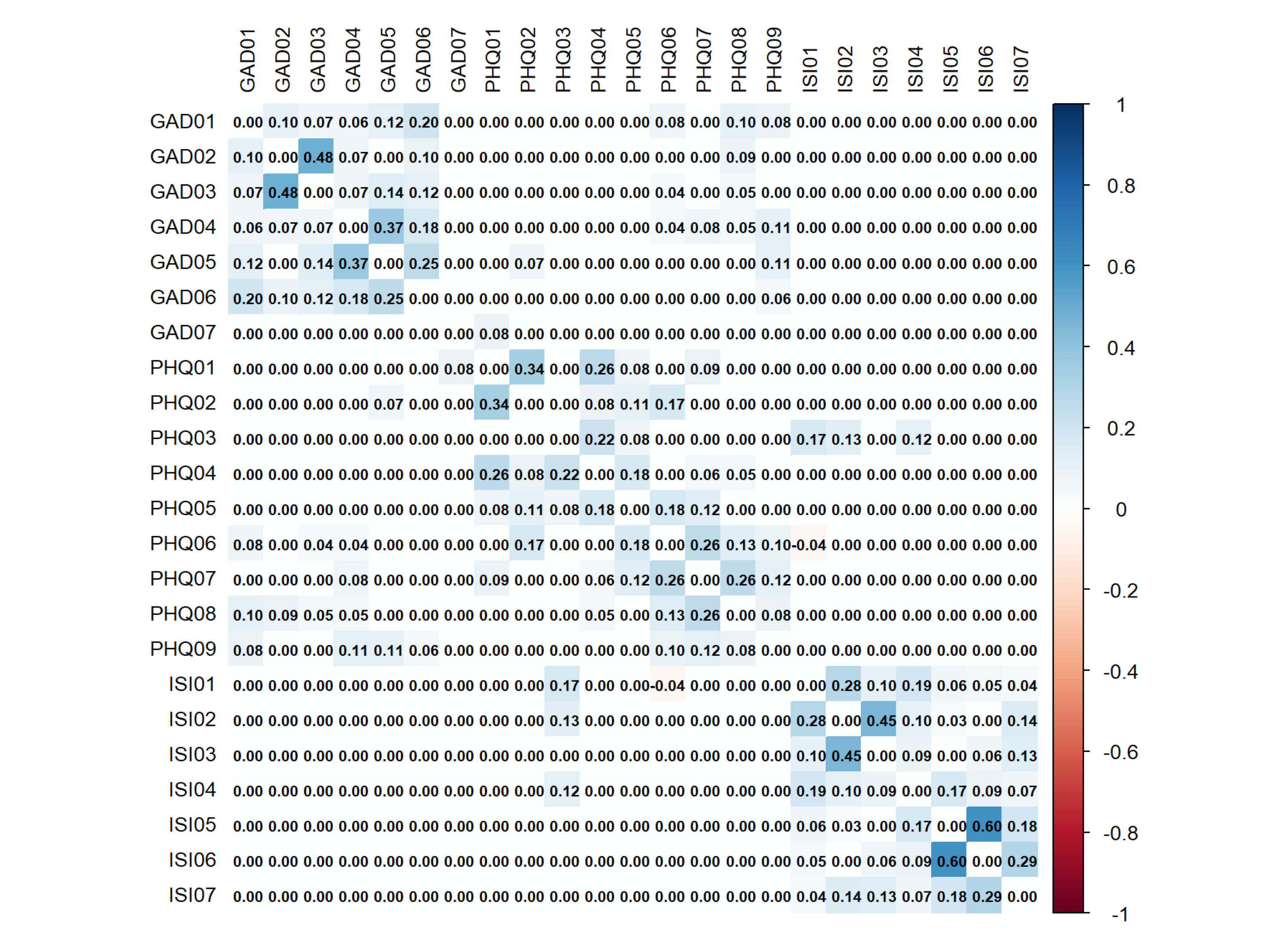

Supplement: Supplemental Information 5 [file peerj-14-20868-s005.png]

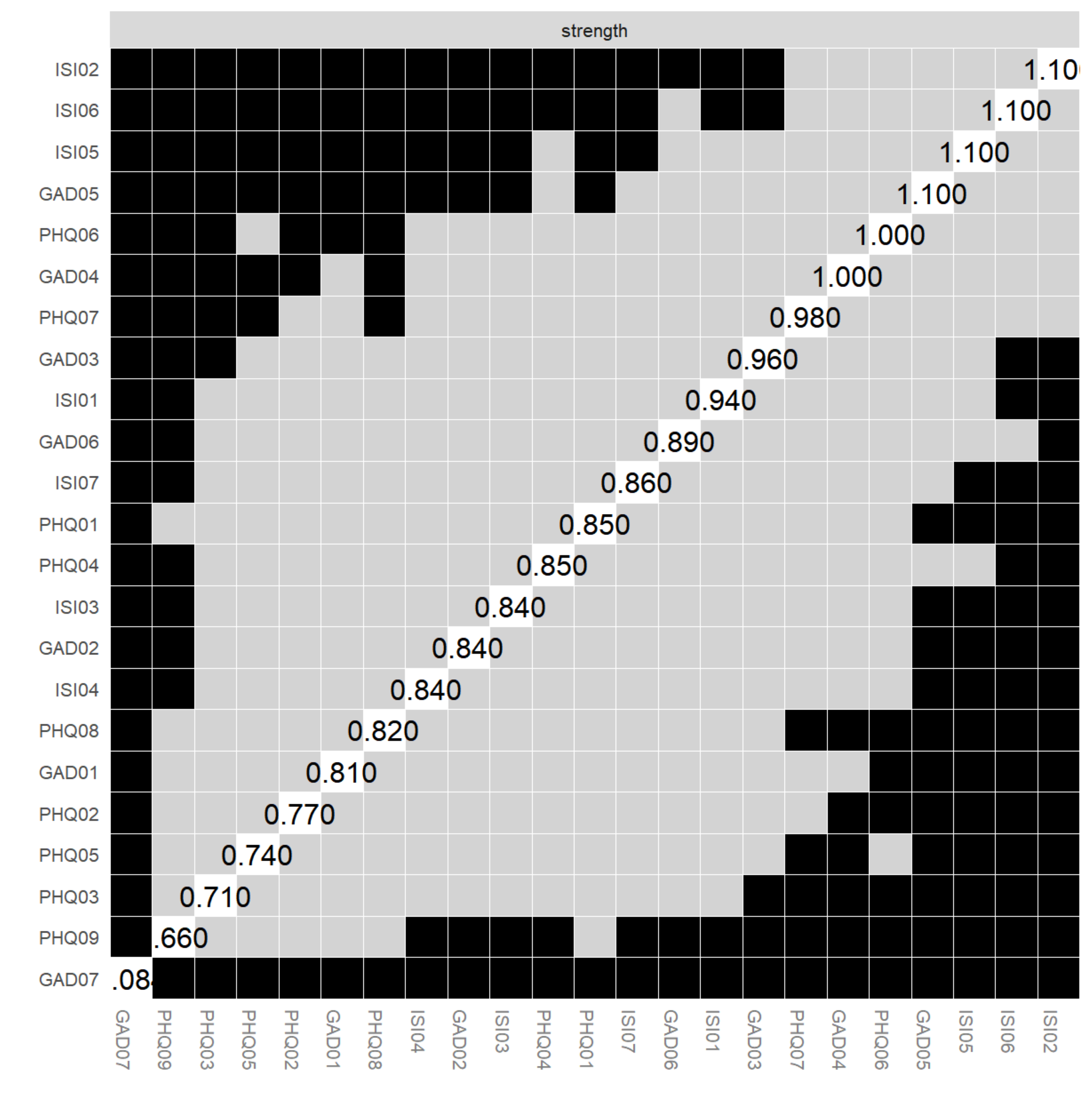

Supplement: Supplemental Information 7 [file peerj-14-20868-s007.png]

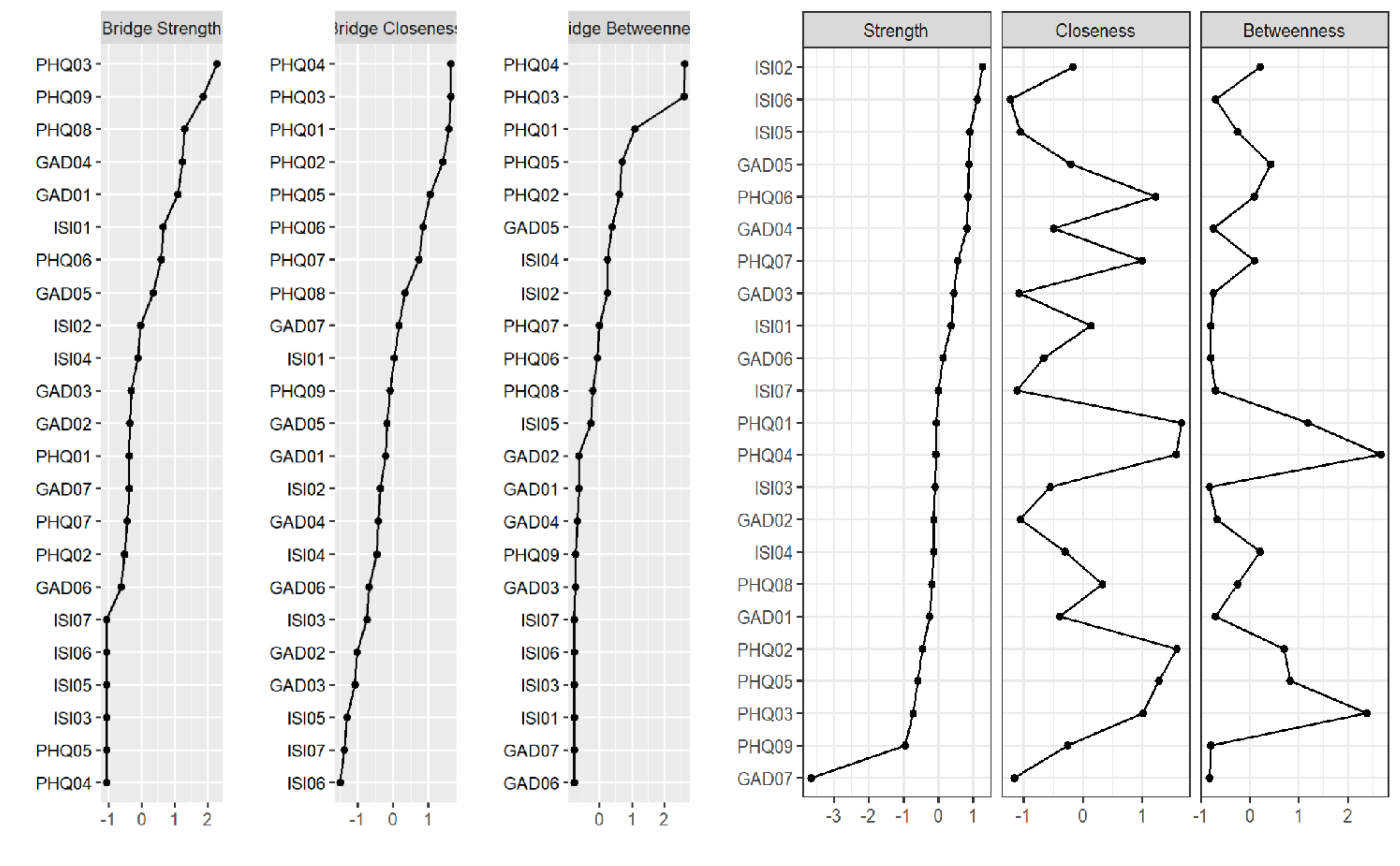

Supplement: Supplemental Information 9 [file peerj-14-20868-s009.png]
