# Supplementary material for: Network analysis of anxiety, depression and insomnia in the elderly in Jiangsu Province
Source: PeerJ. 2026 Mar 31;14:e20868. doi: 10.7717/peerj.20868 (PMC13048223; doi:10.7717/peerj.20868)
Supplement: Supplemental Information 10 [file peerj-14-20868-s010.docx]

 A) Network_Analysis_code.R

| **Original text (Chinese)** | **English translation** | **Purpose** |
| --- | --- | --- |
| 身份证号码 | Participant ID | Unique identifier for each participant |
| 计算 PHQ01 到 PHQ09 列的和并存储在新列 PHQ_Score 中 | Calculate the sum of columns PHQ01 to PHQ09 and store the result in the new column PHQ_Score. | Function of the code |
| 提取指定列到新的数据框 data2 | Extract the specified columns into a new data frame named data2 | Function of the code |
| D:/我要毕业/江苏省老年人调查问卷下载/江苏.xlsx | D:/My Graduation Project/Jiangsu Province Elderly Population Survey Questionnaire Download/Jiangsu.xlsx | Data file path |

B) GAD+PHQ.xlsx

| **Original text (Chinese)** | **English translation** |
| --- | --- |
| 对做事情缺乏兴趣或乐趣 | Lack of interest or pleasure in doing things |
| 感到心情低落、沮丧或绝望 | Feeling down, depressed, or hopeless |
| 入睡困难、早醒或睡得过多（超过 10 小时） | Trouble falling asleep, waking up too early, or sleeping too much (over 10 hours) |
| 感到疲倦或没什么精力 | Feeling tired or having little energy |
| 食欲不振或暴饮暴食 | Poor appetite or overeating |
| 对自己感到自责，觉得自己是失败者或辜负了家人 | Feeling bad about yourself or that you are a failure or let your family down |
| 难以集中注意力，例如读书、看电视或做家务时分心 | Trouble concentrating on things, such as reading, watching TV, or doing chores |
| 动作或说话变慢，或坐立不安、烦躁不安 | Moving or speaking slowly, or being fidgety/restless |
| 有“死了会更好”或自伤的想法，但未必付诸行动 | Thoughts that you would be better off dead or self-harm thoughts, without necessarily acting on them |
| 感到紧张、焦虑或坐立不安，甚至易怒 | Feeling nervous, anxious, or on edge, even losing temper |
| 对各种事情过度担忧，无论大小、相关与否 | Excessively worrying about various things, big or small, related or unrelated |
| 无法停止或控制对上述事情的担忧 | Unable to stop or control worrying about the above matters |
| 总是感到紧张，很难放松 | Feeling constantly tense, finding it hard to relax |
| 因紧张或烦躁难以安坐或放松 | Difficulty sitting still or relaxing due to tension or restlessness |
| 容易生气或烦躁 | Becoming easily irritated or annoyed |
| 对爱好或活动失去兴趣；参与度下降（例如整天躺着看电视、避免外出、宅在家里、活动少、感到没精力） | Losing interest in hobbies or activities; reduced engagement (e.g., lying around watching TV all day, avoiding going out, staying home, low physical activity, feeling lethargic) |

ISI.xlsx

| **Original text (Chinese)** | **English translation** |
| --- | --- |
| 过去两周失眠的严重程度 | Insomnia severity in the past two weeks (0 = none, 4 = very severe) |
| 对自己睡眠的满意程度 | How satisfied are you with your sleep? (0 = very satisfied, 4 = very dissatisfied) |
| 睡眠问题对日常生活的影响（如白天疲劳、工作或家务、注意力、记忆、情绪等） | How much do your sleep problems affect your daily life? (e.g., daytime fatigue, work or chores, attention, memory, mood) (0 = no effect, 4 = obvious effect) |
| 相比其他降低生活质量的因素，睡眠问题是否更突出 | Are your sleep problems more significant compared to other factors reducing your quality of life? (0 = not obvious, 4 = very obvious) |
| 对当前睡眠问题是否感到焦虑或担忧 | Are you anxious or worried about your current sleep problems? (0 = not at all, 4 = very worried) |

C) sociodemographic_characteristics.xlsx.

| **Variable** | **Description** | **Values / Coding** |
| --- | --- | --- |
| ID | Participant ID | Unique numeric ID |
| AGE | Age in years | Continuous numeric value |
| SEX | Biological sex | 1 = Male; 2 = Female |
| MAR | Marital status | 1 = Married; 2 = Unmarried / Cohabiting; 3 = Widowed; 4 = Divorced |
| EDU | Education level | 1 = Illiterate / Semi-illiterate / Primary school; 2 = Middle school / Technical school; 3 = High school / College / University / Master |
| GEO | Current residential area | 1 = Suburban; 2 = Urban |
| FMI | Monthly family income | 1 = <3000; 2 = 3000–6000; 3 = >6000 |
| EP | Economic pressure | 0 = None; 1 = Some / Small / High / Very high |
| NOC | Number of children | 0 = No children; 1 = ≥1 children |
| OCC | Occupation status | 1 = Retired / Formerly retired; 2 = Rehired; 3 = Re-employed; 4 = Other |
| SMO | Smoking status | 1 = Never; 2 = Quit; 3 = Current smoker |
| DRI | Drinking status | 1 = Never / occasional; 2 = Former drinker; 3 = Current drinker |
| XG | History of COVID-19 infection | 0 = No; 1 = Yes |
| SLE | Average sleep duration per night | 1 = <5 h; 2 = 5–7 h; 3 = 7–9 h; 4 = >9 h |
| GZ | Bone fracture in past year | 0 = No; 1 = Yes |
| LWS | Living alone | 0 = Alone; 1 = With others |
| SAT | Satisfaction with housing and environment | 0 = No; 1 = Yes |
| INT | Having fixed hobbies/interests | 0 = No; 1 = Yes |
| SOC | Participation in social activities | 0 = No; 1 = Yes |
| GAD | Anxiety status | 0 = No; 1 = Yes |
| ZZHD | Ability of independent activity | 0 = No, 1 = Yes |
| MNA | Nutritional status | 0 = Poor nutrition, 1 = Good nutrition |
| PHQ | Depression status | 0 = No; 1 = Yes |
| GAD01 | GAD-7 Item 1 | Continuous numeric value |
| GAD02 | GAD-7 Item 2 | Continuous numeric value |
| GAD03 | GAD-7 Item 3 | Continuous numeric value |
| GAD04 | GAD-7 Item 4 | Continuous numeric value |
| GAD05 | GAD-7 Item 5 | Continuous numeric value |
| GAD06 | GAD-7 Item 6: | Continuous numeric value |
| GAD07 | GAD-7 Item 7 | Continuous numeric value |
| PHQ01 | PHQ-9 Item 1 | Continuous numeric value |
| PHQ02 | PHQ-9 Item 2 | Continuous numeric value |
| PHQ03 | PHQ-9 Item 3 | Continuous numeric value |
| PHQ04 | PHQ-9 Item 4 | Continuous numeric value |
| PHQ05 | PHQ-9 Item 5 | Continuous numeric value |
| PHQ06 | PHQ-9 Item 6 | Continuous numeric value |
| PHQ07 | PHQ-9 Item 7 | Continuous numeric value |
| PHQ08 | PHQ-9 Item 8 | Continuous numeric value |
| PHQ09 | PHQ-9 Item 9 | Continuous numeric value |
| ISI01 | ISI Item 1 | Continuous numeric value |
| ISI02 | ISI Item 2 | Continuous numeric value |
| ISI03 | ISI Item 3 | Continuous numeric value |
| ISI04 | ISI Item 4 | Continuous numeric value |
| ISI05 | ISI Item 5 | Continuous numeric value |
| ISI06 | ISI Item 6 | Continuous numeric value |
| ISI07 | ISI Item 7 | Continuous numeric value |
| ISI_Score | ISI total | Sum of ISI01–ISI07 |
| BMI | Body Mass Index | Continuous numeric value |
